# Upregulation of the Cell-Cycle Regulator RGC-32 in Epstein-Barr Virus-Immortalized Cells

**DOI:** 10.1371/journal.pone.0028638

**Published:** 2011-12-06

**Authors:** Sandra N. Schlick, C. David Wood, Andrea Gunnell, Helen M. Webb, Sarika Khasnis, Aloys Schepers, Michelle J. West

**Affiliations:** School of Life Sciences, University of Sussex, Falmer, Brighton, United Kingdom; Duke University Medical Center, United States of America

## Abstract

Epstein-Barr virus (EBV) is implicated in the pathogenesis of multiple human tumours of lymphoid and epithelial origin. The virus infects and immortalizes B cells establishing a persistent latent infection characterized by varying patterns of EBV latent gene expression (latency 0, I, II and III). The CDK1 activator, Response Gene to Complement-32 (RGC-32, C13ORF15), is overexpressed in colon, breast and ovarian cancer tissues and we have detected selective high-level RGC-32 protein expression in EBV-immortalized latency III cells. Significantly, we show that overexpression of RGC-32 in B cells is sufficient to disrupt G2 cell-cycle arrest consistent with activation of CDK1, implicating RGC-32 in the EBV transformation process. Surprisingly, RGC-32 mRNA is expressed at high levels in latency I Burkitt's lymphoma (BL) cells and in some EBV-negative BL cell-lines, although RGC-32 protein expression is not detectable. We show that RGC-32 mRNA expression is elevated in latency I cells due to transcriptional activation by high levels of the differentially expressed RUNX1c transcription factor. We found that proteosomal degradation or blocked cytoplasmic export of the RGC-32 message were not responsible for the lack of RGC-32 protein expression in latency I cells. Significantly, analysis of the ribosomal association of the RGC-32 mRNA in latency I and latency III cells revealed that RGC-32 transcripts were associated with multiple ribosomes in both cell-types implicating post-initiation translational repression mechanisms in the block to RGC-32 protein production in latency I cells. In summary, our results are the first to demonstrate RGC-32 protein upregulation in cells transformed by a human tumour virus and to identify post-initiation translational mechanisms as an expression control point for this key cell-cycle regulator.

## Introduction

Epstein-Barr virus (EBV) is a human gamma herpes virus carried by greater than 90% of the world's population as a largely asymptomatic persistent latent infection in B-lymphocytes. Despite the fact that EBV-infected cells proliferate indefinitely [1], effective immune control usually prevents tumour outgrowth in healthy hosts. EBV has however been shown to contribute to the development of numerous human cancers e.g. Burkitt's lymphoma, undifferentiated nasopharyngeal carcinoma, Hodgkin's disease and AIDS-associated and transplant-associated immunoblastic lymphomas (reviewed in [2]). Immortalization of resting B cells by EBV *in vitro* leads to the generation of latently infected lymphoblastoid cell lines (LCLs) that express all EBV latent proteins: Epstein-Barr nuclear antigens (EBNAs) 1, 2, 3A, 3B, 3C, -LP and Latent membrane proteins (LMPs) 1, 2A and 2B, in addition to non-coding RNA species. This ‘full’ pattern of latent gene expression is termed latency III. More restricted patterns of latent gene expression were first detected in tumour cells; EBV-positive Burkitt's lymphoma (BL) cells express only one latent antigen, EBNA 1 (latency I), where the malignant cells of Nasopharyngeal carcinomas and Hodgkin lymphomas express the LMPs in addition to EBNA1 (Latency II). Since the latency III pattern of gene expression is only associated with EBV positive tumours arising in immunosuppressed post-transplant or AIDS patients, it appeared that latent gene expression was downregulated during tumourigenesis as part of an immune-evasion strategy. However, latency I and II phenotypes were subsequently detected in healthy EBV-infected individuals indicating that EBV positive cells display different patterns of latent gene expression during the establishment of a persistent infection, raising the possibility that the latency type of tumour cells may simply reflect that of the precursor cell [3]-[4]. Non-dividing EBV-positive cells lacking any latent gene expression have also been detected in infected hosts (latency 0), demonstrating that infected cells can ‘shut-off’ latent gene expression when in a resting state [3].

EBV has the capacity to disrupt the G1/S, G2/M and mitotic cell-cycle checkpoints, thus promoting the proliferation of infected cells to facilitate the establishment of a persistent viral infection in the host. Studies examining the G1/S checkpoint in primary B cells infected with EBV *in vitro* have demonstrated that treatment with genotoxins that induce the formation of adducts and cross-links results in normal stabilisation and activation of p53 but the cyclin-dependent kinase inhibitor (CDKI) p21^WAF1/CIP1^ fails to accumulate. As a result CDK2 remains active and cells can progress into S phase with damaged DNA [5]-[6]. Interestingly, the response of these cells to DNA damage in the form of double-strand DNA breaks appears to differ and both p53 and p21^WAF1/CIP1^ responses are maintained, indicating that EBV modulates the response to different types of damage in different ways [5]-[7]. Studies into the effects of EBV on the G2/M checkpoint have demonstrated that although EBV-negative Burkitt's lymphoma cells treated with genotoxins arrest in G2/M, EBV-infected derivatives of these cells continue to progress through G2/M and are protected from apoptosis [8]. EBV-positive cells are also able overcome G2 arrest induced by a histone deacetylase inhibitor [9]. EBV infection of BL lines additionally promotes survival following induction of the mitotic spindle checkpoint by microtubule destabilising drugs through both checkpoint disruption and reduced cell death mediated by downregulation of the proapoptotic protein, Bim [10].

The essential latency III protein, EBNA 3C, has emerged as a key player in EBV-mediated cell-cycle disruption; when expressed alone in various cell-types EBNA 3C has the capacity to disrupt the G1/S, G2/M and mitotic checkpoints [9], [11]–[12]. Moreover, EBNA 3C inactivation halts the growth of EBV-infected cells as a result of transcriptional derepression of the CDKI p16^INK4a^ [13]–[14]. Interestingly, cooperation between EBNA 3C and another member of the EBNA 3 family, EBNA 3A has been shown to be required for both repression of Bim and repression of p16^INK4a^ through an epigenetic mechanism involving increased trimethylation of Histone H3 on lysine 27 at the gene loci [15]–[16]. There have also been reports describing interactions between EBNA 3C and multiple cell-cycle regulatory proteins including cyclin A, cyclin D1, SCFSkp2, Rb, c-Myc, chk2, p53, Mdm2 and the p53 regulatory proteins ING4 and 5 [17]–[26].

Response Gene to Complement 32 (RGC-32, C13ORF15) was identified in rat oligodendrocytes as a novel gene induced in response to sub-lytic complement activation of the cell cycle [27]. Cloning of human RGC-32 cDNA from a foetal brain library identified a cDNA encoding a protein of 117 amino acids with no significant primary sequence homology to other human proteins (AF036549) [28]. RGC-32 was shown to be expressed at the RNA and protein level in a range of human tissues including brain, heart and liver [28]. Consistent with a role in cell-cycle progression, expression of RGC-32 in smooth muscle cells following G1 arrest promotes S- and M-phase entry and RGC-32 has been shown to bind and activate the key mitotic kinase, CDK1, in a manner dependent on threonine 91 phosphorylation of RGC-32 by CDK1 [27]–[28]. Moreover, knock-down of RGC-32 prevents complement and growth factor-induced cell-cycle entry and CDK1 activation in aortic endothelial cells [29]. RGC-32 translocates from the cytoplasm to the nucleus during cell-cycle activation or the onset of mitosis and associates with centrosomes [28], [30]. Interestingly, RGC-32 has been shown to be overexpressed at the RNA and/or protein level in multiple human tumours including those of the colon, prostate, bladder, breast, lung and ovaries [31]–[33]. High-level RGC-32 expression appears to correlate with late stage disease, since increased RGC-32 RNA and protein levels are detected in advanced stages of colon carcinoma, compared to precancerous or early stage colon cancer tissues [34].

Although much evidence points to a role for RGC-32 in the promotion of cell proliferation, some studies have implicated RGC-32 as a tumour suppressor. The RGC-32 gene was found to be frequently inactivated in glioma cell-lines, with RGC-32 expression levels correlating with the p53 status of the cell-lines [30]. Further analysis revealed RGC-32 as a transcriptional target of p53 [30]. The same study demonstrated that overexpression of RGC-32 delayed progress through mitosis and suppressed the growth of glioma cells, indicative of a negative effect on cell growth. Microarray profiling of multiple myeloma plasma cells and drug resistant glioblastomas has also detected underexpression of RGC-32 mRNA [35]–[36] implicating downregulation of RGC-32 in tumour development/progression. Recently, methylation of the RGC-32 promoter region was associated with RGC-32 downregulation in non small cell lung cancers [37]. Since a number of regulatory pathways have been reported to control RGC-32 expression in a variety of cellular systems including TGF-β signalling in neural crest cells and the MAPK pathway in aortic and umbilical vein endothelial cells, it is likely that multiple mechanisms may act to fine-tune RGC-32 expression in a cell-type-specific manner (reviewed in [38]). Accumulating evidence indicates that perturbation of these control mechanisms may play a key role in the development of a diverse range of human cancers.

We have detected high-level RGC-32 protein expression in Epstein-Barr virus-infected B-cells and provide the first demonstration that RGC-32 overexpression disrupts G2/M checkpoint regulation, implicating RGC-32 in the EBV transformation mechanism. We have identified key new control mechanisms for the regulation of RGC-32 expression in EBV-infected human B-cells. We show that RGC-32 mRNA expression is controlled by the RUNX1c transcription factor leading to high level RGC-32 mRNA expression in cells displaying the restricted latency I form of EBV gene expression. However, our data indicate that a post-initiation translational block prevents RGC-32 protein expression in EBV negative and latency I cells.

## Results

### RGC-32 protein is differentially upregulated in EBV-positive cell-lines

**Figure 1:**
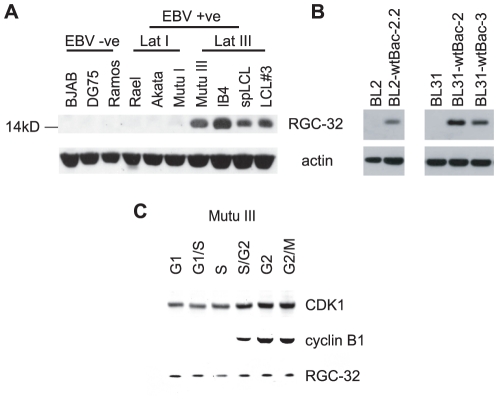
RGC-32 protein is selectively expressed in EBV infected latency III cells. (A) Western blot analysis of RGC-32 protein expression in a panel of EBV negative and positive B-cell-lines. Blots were stripped and re-probed with anti-actin antibodies as a loading control (B) Western blot analysis of RGC-32 protein expression in two EBV negative BL cell-lines (BL2 and BL31) infected with recombinant wild-type EBV bacmids [15]. (C) Western blot analysis of RGC-32 protein expression in Mutu III cell-cycle fractions obtained by centrifugal elutriation. Blots were probed for CDK1 and cyclin B1 as controls for cell-cycle phases. Cell-cycle phases were confirmed by Flow cytometry (Figure S1).

Examination of RGC-32 expression in a panel of EBV-negative and positive cell-lines revealed that RGC-32 protein was highly expressed in EBV-infected lymphoblastoid cell-lines that express the ‘full’ panel of latent gene expression, termed latency III (Figure 1A). We also detected RGC-32 protein expression in a latency III Burkitt's lymphoma (BL) cell-line clone (Mutu III) that had drifted in culture from an original BL line (Mutu I) displaying the characteristic more restricted form of EBV gene expression, latency I (EBNA 1 only) [39] (Figure 1A). In contrast, we were unable to detect RGC-32 protein expression in all EBV negative and EBV-positive latency I B-cell lines examined (Figure 1A and data not shown). Moreover, RGC-32 protein expression was induced on infection of two EBV negative BL cell-lines, BL2 and BL31 with a recombinant EBV bacmid [15] (Figure 1B). Since upregulation of RGC-32 expression has been linked to numerous cancers [31]–[34], potentially through the role of RGC-32 as an activator of the mitotic CDK1/cyclin B1 complex [27]–[28], these results may implicate RGC-32 upregulation in EBV-mediated tumourigenesis. To determine whether levels of RGC-32 fluctuated during the cell cycle, we examined RGC-32 protein expression in cell-cycle fractions obtained by centrifugal elutriation from Mutu III cells (Figure 1C). Our results demonstrated that total RGC-32 protein levels do not vary significantly during the cell-cycle, indicating that any cell-cycle specific effects of RGC-32 are likely to be mediated through control of RGC-32 activity or cell-cycle specific expression of RGC-32 targets.

### Stable overexpression of RGC-32 alone is sufficient to disrupt the G2/M checkpoint

**Figure 2:**
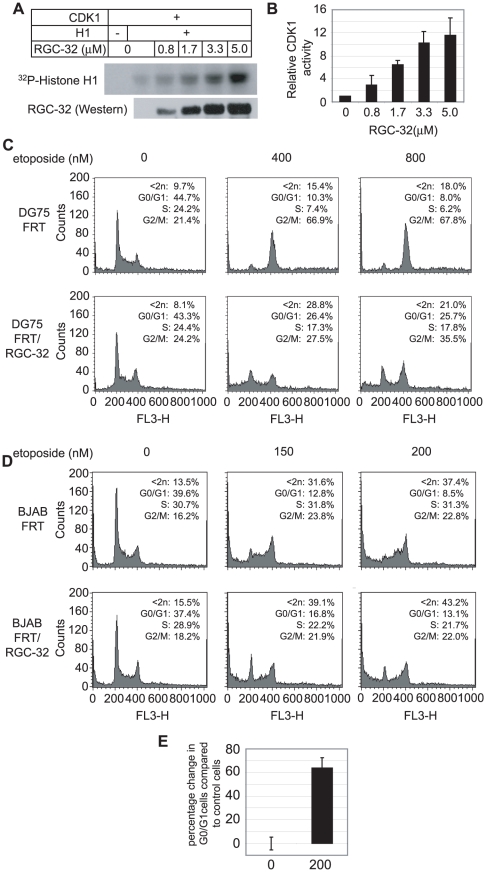
RGC-32 activates CDK1 and disrupts the G2/M checkpoint in B-cell lines. (A) Histone H1 kinase assay carried out using recombinant CDK1/Cyclin B1 in the absence or presence of purified recombinant His-RGC-32. (B) Quantification of [^32^P]-Histone H1 signals. Results show mean fold increases in CDK1 activity in the presence of RGC-32 +/- standard deviation from 3 independent experiments. (C) Representative cell cycle profile analysis of DG75 FRT control cells and DG75 FRT/FLAG-RGC-32 cells treated for 24 hours with etoposide. Control cells (0) were harvested prior to etoposide treatment. (D) Representative cell cycle profile analysis of BJAB FRT control cells and BJAB FRT/FLAG-RGC-32 cells treated for 48 hours with etoposide. (E) Graph showing the percentage change in the G0/G1 population in BJAB FRT/FLAG-RGC-32 cells compared to control BJAB FRT cells, prior to (0), or following etoposide treatment for 48 hours. Results represent the mean +/− standard deviation of 3 independent experiments.

To confirm the effects of RGC-32 on CDK1 activity we carried out Histone H1 kinase assays using purified recombinant His-tagged RGC-32 and observed a dose-dependent increase in CDK1 activity in the presence of RGC-32 (Figure 2A). In replicate kinase assays containing 5 µM His-RGC-32, CDK1 activity was enhanced by an average of 11.6-fold (Figure 2B).

CDK1 is held in an inactive tyrosine 15 and threonine 14 phosphorylated form during interphase and is activated through dephosphorylation by the cdc25 phosphatase at the end of G2, allowing entry into mitosis. When the G2/M checkpoint is triggered on exposure to DNA damaging agents, CDK1 activation is prevented through the phosphorylation, sequestration and degradation of cdc25, resulting in G2 arrest. Since we had confirmed that RGC-32 could function as an activator of CDK1 (Figure 2A and B), we next examined whether overexpression of RGC-32 alone was sufficient to disrupt the G2/M checkpoint in B-cell lines. We created isogenic EBV negative B cell-lines (DG75 and BJAB) stably expressing Flag-tagged RGC-32 (FRT/RGC-32) using the Flp-in system (Invitrogen). Control cell-lines and FRT/RGC-32 cell-lines were then treated with the topoisomerase II inhibitor, etoposide, to introduce DNA double strand breaks and trigger the G2/M checkpoint. Our results demonstrated that in contrast to DG75 FRT control cells that accumulated in G2/M following etoposide treatment, DG75 FRT/RGC-32 cells displayed a higher proportion of cells in G0/G1 and reduced levels of cells in G2/M indicating transit of a significant proportion of cells through the checkpoint (Figure 2D). We observed a 2.6 and 3.2-fold increase in the proportion of cells in G0/G1 in DG75 FRT/RGC-32 cells compared to control cells following treatment for 24 hrs with 400 nM and 800 nM etoposide, respectively. RGC-32 expressing DG75 cells also displayed 60% and 52% decreases in the G2/M population at these concentrations of etoposide, compared to control cells. BJAB cells appeared to tolerate the FRT expression system less well than DG75 cells, with increased sub 2n populations in both control FRT cells and FRT/RGC-32 cells (Figure 2E). These cell-lines also displayed more sensitivity to etoposide treatment and phenotypes were most evident when cells were treated with lower etoposide concentrations for a longer time (48 hrs). Nonetheless, clear differences were observed in the cell-cycle distribution of BJAB FRT/RGC-32 cells compared to control FRT cells in response to etoposide, with increased numbers of cells in G0/G1 indicating increased passage of cells through the G2/M checkpoint (Figure 2E). Interestingly, in the BJAB FRT background, checkpoint disruption was represented by a phenotype of a decreased S-phase population and an increased G0/G1 population, with only a slight decrease in the G2/M population. This observation is likely to result from the fact that control BJAB/FRT cells appear to be arrested in both S and G2 phases, but in BJAB/RGC-32 cells both S phase and G2 cells transit through the G2/M checkpoint and either die or re-enter G1. Consistent with checkpoint transit, an increase in the G0/G1 population was observed in multiple etoposide experiments carried out in BJAB/RGC-32 cells (Figure 2F). In both DG75 and BJAB cell backgrounds, transit through the G2/M checkpoint in etoposide-treated RGC-32 expressing cells also led to increases in the sub 2n population indicative of the apoptotic death of some cells unable to repair damaged DNA (Figure 2 C and D). In summary, our results provide the first evidence that overexpression of RGC-32 in two different B-cell backgrounds can disrupt the G2/M checkpoint and suggest that upregulation of RGC-32 could play a role in EBV-mediated cell-cycle deregulation in B-cells.

### RGC-32 mRNA expression is highest in EBV positive latency I cells

**Figure 3:**
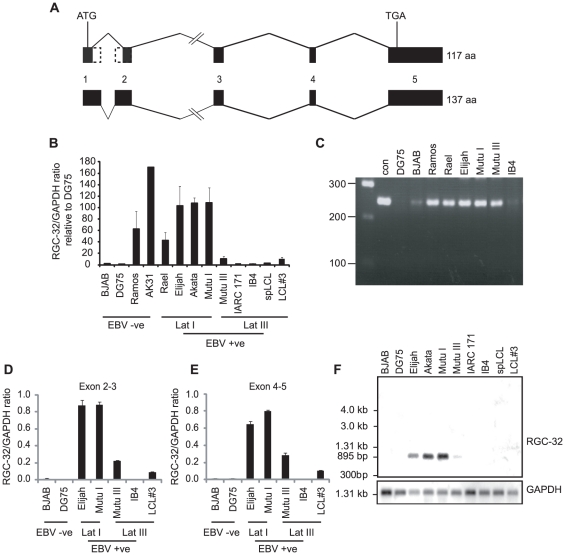
RGC-32 mRNA expression is differentially regulated in different types of EBV latency. (A) Diagram showing RGC-32 transcript variants. Black boxes represent exons and dotted lines show the parts of exons 1 and 2 that are not included in the shorter RGC-32 transcript (AF036549) that encodes a protein of 117 amino acids [28]. The longer RGC-32 (C13ORF15) transcript (NM_014059) is predicted to encode a protein of 137 amino acids. (B) Q-PCR analysis using primers in exon 3. Transcript quantities were normalized to those of GAPDH and then expressed relative to the signal obtained in DG75 cells. Results show the mean of 3 independent experiments +/− standard deviation. (C) Non-quantitative PCR analysis of cDNA samples using primers that amplify across exon 2 to exon 4. pFLAG-RGC-32 was used as a positive control (con). Q-PCR analysis using primers that amplify across exons 2 and 3 (D) or exons 4 and 5 (E). Transcript quantities were normalized to GAPDH and results show the mean of 3 independent experiments +/− standard deviation. (F) Northern blot analysis of RGC-32 transcripts. Blots were probed with a [^32^P]-labelled probe generated from the entire RGC-32 cDNA and then stripped and re-probed for GAPDH.

In follow-up experiments investigating the expression of RGC-32 mRNA in a panel of EBV negative and positive B-cell lines, we observed that RGC-32 mRNA expression was significantly higher in latency I BL lines, and in some EBV-negative cell-lines, compared to latency III cell-lines (Figure 3B). This was surprising given that no RGC-32 protein expression is detectable in latency I and EBV-negative cell-lines (Figure 1).

Two transcript variants of human RGC-32 have been documented (Figure 3A). The shorter form, previously detected in many cell types, lacks the end of exon 1 and the start of exon 2 [28](Figure 1). Database entries also document a longer form of RGC-32, generated from alternative splice sites at the end of exon 1 and the start of exon 2, that encodes a protein with an additional 20 amino acids close to the N terminus (e.g. NM_014059). Although the Q-PCR primers we used to detect RGC-32 mRNA were within an exon region common to both transcript variants (exon 3), we carried out further analysis to rule out the possibly that these primers were failing to detect a novel alternatively spliced RGC-32 transcript in latency III cells that lacks exon 3. Conventional non-quantitative PCR across exons 2 to 4 produced a single product in EBV-negative and EBV-positive latency I and III cells that was consistent with the size expected if exon 3 were present (248 bp) (Figure 3C). No shorter products indicating the absence of exon 3 (140 bp) were detected (Figure 3C). Further Q-PCR analysis using primers across exons 2 to 3 (Figure 3D) and exons 4-5 (Figure 3E) also confirmed our original observations that latency I cells express high levels of RGC-32 mRNA compared to latency III cells. By way of final confirmation of this unexpected mRNA expression profile, we also investigated the possibility that our RGC-32-specific primers were detecting an additional previously undocumented gene transcript in latency I cells that spans the RGC-32 gene locus and utilizes common exons. Northern blotting analysis using a probe generated from the entire RGC-32 cDNA sequence verified that the only transcript detectable in latency I cells had an approximate size of 900 nts, consistent with the expression of the short-form of RGC-32. In line with the lower sensitivity of Northern blotting vs Q-PCR, RGC-32 transcripts were detectable in latency I cells, were reduced in Mutu III latency III cells and were not detectable in LCLs.

Thus, using multiple approaches, we have demonstrated that RGC-32 mRNA is expressed at high levels in EBV positive latency I cells and some EBV negative cell lines despite a lack of RGC-32 protein expression, implicating post-transcriptional mechanisms in the control of RGC-32 gene expression. It is clear however, that the seemingly low levels of RGC-32 message expressed in latency III cells are sufficient to support robust RGC-32 protein expression in these cells (Figure 1).

### RGC-32 mRNA expression in human B-cells is controlled by RUNX1c

The differential RGC-32 mRNA expression we detected in EBV positive cells resembles that previously documented for the B-cell isoform of the RUNX1 transcription factor, RUNX1c [40]-[41]. Latency-type dependent expression of RUNX1c results from upregulation of the related RUNX3 transcription factor by EBNA 2 in latency III cells [40]. This leads to downregulation of RUNX1 expression, since RUNX3 directly represses RUNX1c transcription by binding to RUNX sites close to the RUNX1 P1 promoter [41]. RUNX1c expression is therefore high in latency I and low in latency III cells. Consistent with the possibility that RUNX1 may regulate RGC-32 transcription in human B-cells, previous reports demonstrated that knockdown of rat RUNX1 resulted in reduced RGC-32 mRNA expression in rat periovulatory cells. RUNX binding sites in the rat RGC-32 promoter were subsequently identified [42]–[43]. Further studies also identified mouse RGC-32 as a direct target of the RUNX 1, 2 and 3 transcription factors when these proteins were overexpressed in fibroblasts [44].

**Figure 4:**
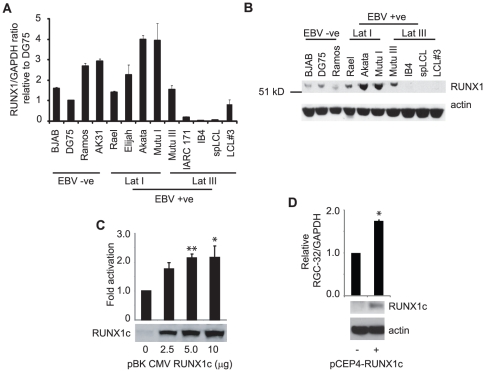
RGC-32 is transcriptionally regulated by RUNX1c in B-cells. (A) Q-PCR analysis of RUNX1c mRNA levels. Transcript quantities were normalized to those of GAPDH and then expressed relative to the signal obtained in DG75 cells. Results show the mean of 3 independent experiments +/− standard deviation. (B) Western blot analysis of RUNX1c protein expression in a panel of EBV negative and positive B-cell-lines. Actin levels serve as a loading control. (C) Transient reporter assays in DG75 cells transfected with 4 µg of the RGC-32 promoter-reporter construct (pRGC-32pluc), 2 µg pRL-CMV as a transfection control and increasing amounts (2.5, 5 and 10 µg) of a RUNX1c-expressing plasmid (pBK-CMV-RUNX1c). Firefly luciferase signals were normalized to Renilla luciferase signals. Results show the mean of 3 independent experiments +/− standard deviation. RGC-32 promoter activation is expressed relative to the RUNX1-negative control. ** P value <0.01 (0.004), * p value <0.05 (0.036). (D) IB4 cells were transfected with pCEP4 or pCEP4-RUNX1c plasmids and transfected cells selected in Hygromycin B. 6 days post-transfection samples were analysed for RUNX1c protein expression by western blotting using actin as a loading control and endogenous RGC-32 mRNA expression using Q-PCR. Results show the mean of 2 independent experiments −/+ standard deviation. * P value <0.05 (0.012).

Real-time PCR analysis of RUNX1c transcript levels and Western blot analysis of RUNX1 protein expression in a panel of EBV-negative and EBV-positive cell-lines confirmed previous reports that high RUNX1 expression correlated with a EBV latency I gene expression pattern [40]-[41]. RGC-32 mRNA expression showed a correlation with RUNX1c expression; with the highest levels detectable in latency I cell lines and some EBV negative cell lines (compare Figure 4A and B with Figure 3B). We next tested whether RUNX1c was able to activate transcription of the human RGC-32 promoter in transient assays using an RGC-32 promoter-reporter construct. We detected a statistically significant greater than 2-fold activation of the RGC-32 promoter by RUNX1c confirming the human RGC-32 promoter as a RUNX1 target (Figure 4C). Consistent with these observations, we found 6 potential RUNX sites in the region of the RGC-32 promoter present in the reporter construct. Further experiments demonstrated that RUNX1c was able to upregulate endogenous RGC-32 transcription. Expression of RUNX1c in an LCL with characteristically low endogenous levels of RUNX1c, followed by short-term drug selection of transfected cells, led to a statistically significant 1.75-fold increase in endogenous RGC-32 mRNA expression 6 days post-transfection (Figure 4D).

### Proteasomal degradation or blocked message export do not prevent RGC-32 protein expression in latency I cells

**Figure 5:**
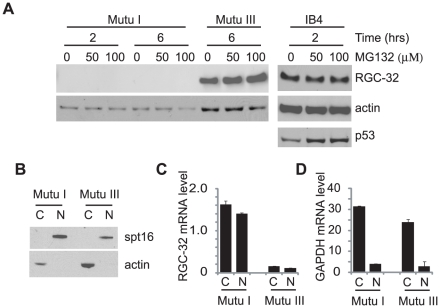
Proteasomal degradation or blocked mRNA export doesn't prevent RGC-32 protein expression in latency I cells. (A) Mutu I, Mutu III and IB4 cells were treated in parallel with 50 or 100 µM MG132 and harvested after 2 or 6 hrs for Western blot analysis. Blots were probed in sections for RGC-32 and actin. IB4 blots were also probed for p53 as a control for MG132-mediated protein stabilization through proteasomal inhibition. (B) Mutu I and III cells were fractionated into cytoplasmic (C) and nuclear (N) protein extracts and analysed by Western blotting for nuclear (Spt16) and cytoplasmic (actin) marker proteins to confirm purity of the fractions. RGC-32 (C) and GAPDH (D) transcript levels were quantified by Q- PCR in cytoplasmic (C) and nuclear (N) RNA extracts prepared in parallel to protein extracts.

To investigate the lack of detectable RGC-32 protein expression in latency I cells further, we examined the possibility that RGC-32 was translated but then rapidly degraded by the proteasome. The rlatency I and latency III BL cell lines Mutu I and Mutu III were treated with the proteasome inhibitor MG132 in parallel with the IB4 LCL (latency III). Treatment with 50 or 100 µM MG132 for up to 6 hrs did not result in the appearance of detectable RGC-32 protein in Mutu I cells (Figure 5A). Parallel treatment of IB4 cells which express functional p53, showed that MG132 inhibition was effective; p53 protein levels increased after MG132 treatment (Figure 5A). MG132 treatment of Mutu III cells appeared to result in some increase in RGC-32 protein levels, although levels of RGC-32 in IB4 cells appeared unaffected. It is therefore possible that the proteasome may play a role in regulating RGC-32 protein expression in some latency III cell backgrounds. We next investigated the possibility that RGC-32 protein was not translated in latency I cells due to a block in the export of RGC-32 message to the cytoplasm. Cytoplasmic and nuclear fractions were prepared from Mutu I and Mutu III cells and protein and RNA extracted in a parallel. Western blotting for a nuclear (Spt16) and cytoplasmic marker (actin) confirmed the integrity of the fractions (Figure 5B). The cytoplasmic and nuclear distribution of RGC-32 mRNA was then determined by Q-PCR (Figure 5C) and compared to that of a control message (GAPDH) that is highly expressed and efficiently exported (Figure 5D). Our results demonstrated that although the proportion of RGC-32 mRNA present in the cytoplasm was lower than that of GAPDH, greater than 50% of the RGC-32 message expressed in latency I cells was detectable in the cytoplasm (Figure 5D) indicating that sufficient RGC-32 mRNA is available in the cytoplasm for translation. These results therefore exclude proteasomal degradation and blocked cytoplasmic export as mechanisms preventing RGC-32 protein detection in latency I cells.

### RGC-32 translation is blocked at a post-initiation stage in latency I cells

**Figure 6:**
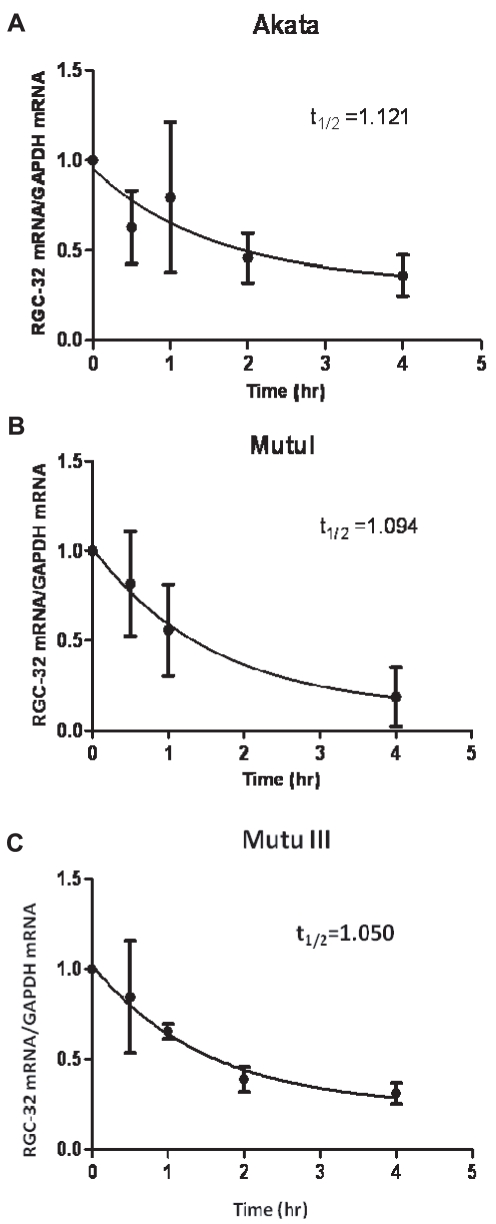
RGC-32 mRNA is not stabilized in latency I cell lines. Cell-lines were treated with Actinomycin D and samples analysed at the times indicated. RGC-32 mRNA levels were deteremined by Q-PCR and normalized to those of the stable control message GAPDH. Results are expressed relative to the level detected at time 0 and show the mean +/− standard deviation of 4 independent experiments for Akata (A) or two independent experiments for Mutu I (B) and Mutu III cells (C). The half-life values indicated were calculated using non-linear regression analysis.

Since our results thus far pointed to a block to RGC-32 translation in EBV-negative and EBV-positive latency I cells, we investigated the possibility that translational repression may result in stabilization of RGC-32 mRNA thus contributing to the high levels of message in these cells. We therefore monitored the rate of degradation of RGC-32 mRNA using Actinomycin D to block transcription. Our results indicated that the rate of degradation of RGC-32 mRNA was similar in the Akata and Mutu I latency I lines compared to the Mutu III latency III cell-line, with the message half-life estimated at approximately 1 hr in all cell-lines (Figure 6). Since a potential translational block in latency I cells does not lead to stabilization of RGC-32 mRNA, high RGC-32 mRNA levels in latency I cells appear to result from transcriptional activation by RUNX1c and potentially other transcription factors expressed in latency I cells (Figure 4).

**Figure 7:**
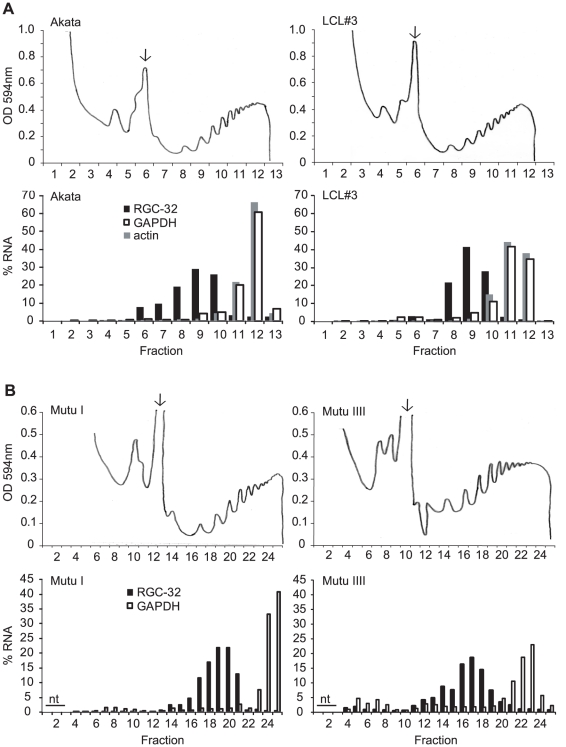
RGC-32 mRNA is associated with polysomes in latency I and latency III cells. Cytoplasmic extracts were sedimented on sucrose density gradients and 0.5 ml or 1 ml fractions collected with continuous monitoring of absorbance at 254 nm (upper panels in A and B). 80S monosome peaks are indicated by arrows. Transcript levels in each fraction were determined using Q-PCR and specific primers to RGC-32 (black bars), GAPDH (open bars) and actin (grey bars). Transcript levels are expressed as a percentage of the total transcript levels detected across the gradient (nt indicates fractions that were not tested). (A) Parallel analysis of Akata (latency I, no RGC-32 protein expression) and LCL#3 (latency III, RGC-32 protein expressed) polysomes. (B) Parallel analysis of Mutu I (latency I, no RGC-32 protein expression) and Mutu III (latency III, RGC-32 protein expressed) polysomes.

To investigate the regulation of RGC-32 translation in latency I and latency III cells, we next investigated whether RGC-32 was associated with polyribsomes (polysomes) in both latency types. Monosomal and polysomal-associated messages were separated using sucrose gradient density centrifugation and the distribution of RGC-32 and control messages in gradient fractions determined using Q-PCR. Surprisingly, parallel analysis carried out in Akata latency I cells and a latency III cell line, LCL#3 revealed that RGC-32 mRNA was associated with polysomes in both cell lines, indicative of association of multiple ribosomes with the RGC-32 message (Figure 7A). Both GAPDH and actin control messages were associated with a high number of ribosomes or ‘heavy’ polysomes in both cell-lines, consistent with their constitutive high-level expression. Similar results were obtained when parallel analysis was carried out in latency I Mutu I cells and latency III Mutu III cells (Figure 7B). Since RGC-32 mRNA is associated with polysomes even when RGC-32 protein is absent in latency I cells, these results indicate that RGC-32 translation is blocked at a post-initiation stage. Post-initiation translational repression can involve reduced translation elongation rates, ribosome drop-off or co-translational protein degradation (involving proteasomal or non- proteasomal pathways). Our data therefore identify a novel post-initiation translational mechanism that blocks RGC-32 protein expression in latency I cells, a mechanism that is presumably relieved in latency III cells.

## Discussion

We have provided the first demonstration that protein expression of the novel CDK1 activator, RGC-32, is upregulated in cells immortalized by a human tumour virus, further supporting a role for deregulated RGC-32 expression in tumour development. Previous studies have described RGC-32 upregulation in ovarian, colon, breast and prostate cancers indicating that deregulation of the cell cycle by RGC-32 may play a key role in the aetiology of a diverse range of tumours [31]–[33]. Since RGC-32 protein expression is not detected in latency I Burkitt's lymphoma cells it is possible that the deregulated expression of c-Myc, resulting from the Myc-Immunoglobulin translocation characteristic of BL, alleviates the requirement for the proliferative advantage potentially provided by RGC-32 protein expression in the EBV transformed LCLs in which it is expressed. Further work is needed to test the hypothesis that RGC-32 protein expression is required for the proliferation of EBV transformed LCLs and RNA interference experiments would be useful in this regard. It is interesting that conversion of latency I BL cells to a latency III EBV gene expression profile as observed in Mutu cells, or infection of EBV negative BL cells is sufficient to ‘re-instate’ RGC-32 protein expression, indicating that latency III gene products play a role in RGC-32 upregulation. Interestingly, other authors have demonstrated that RGC-32 mRNA levels are positively regulated by the EBV-encoded transcriptional regulators EBNA 3A and 3B in LCLs and negatively regulated by EBNA 2 expression in an EBV-negative B-cell-line [45]–[47], implicating interplay between latency III gene products in fine-tuning RGC-32 mRNA expression. These studies however did not address the effects of these changes on RGC-32 protein expression and it is still unclear what factors regulate RGC-32 protein expression in EBV-transformed cells.

Since RGC-32 binds CDK1 *in vitro* and *in vivo* and increases CDK1 activity in kinase assays [27]–[28], the upregulation of RGC-32 in EBV-positive latency III cells led us to investigate whether RGC-32 was a potential mediator of the deregulatory effects of EBV on the G2/M checkpoint. Our data provide the first evidence that overexpression of RGC-32 alone can disrupt the G2/M checkpoint. In support of our observations, inducible overexpression of testis-specific protein Y (TSPY) in HeLa cells has been shown to upregulate RGC-32 and accelerate progression through G2/M [48]. Interestingly, TSPY is located in the Y chromosome gonadoblastoma oncogenic locus and is upregulated in gonadoblastoma, testicular germ-cell tumours, prostate and liver cancers and in melanoma [49]–[52].

Although we confirmed that RGC-32 activates CDK1/cyclin B1 *in vitro*, the mechanism of CDK1 activation by RGC-32 remains to be fully elucidated. Although RGC-32 has no homology to other human proteins, it may be functionally similar to members of the Speedy/RINGO family of novel CDK activators first described as inducers of G2/M progression in *Xenopus* oocytes [53]–[54]. Significantly, overexpression of human speedy 1 promotes G1/S transition and speedy/RINGO C stimulates late S-phase progression and disrupts DNA damage-induced G2 arrest [55]–[56]. RINGO family members have been shown to bind and activate CDKs in the absence of cyclin, through mechanisms that alleviate the requirement for activation by CDK-activating kinase (CAK) and override the effects of inhibitory phosphorylation (for review see [57]). It will be interesting to determine whether RGC-32-mediated activation can be cyclin-independent, override inhibitory phosphorylation and/or promote CDK1 activation through dephosphorylation. Interestingly, Saigusa *et al* showed that RGC-32 interacts with the centrosome-associated polo-like kinase 1 (Plk1) and is phosphorylated by Plk1 *in vitro,* identifying another possible mediator of the effects of RGC-32 on the cell cycle [30].

Surprisingly, Saigusa *et al* also identified RGC-32 as a potential tumour suppressor gene deleted in malignant gliomas that could suppress growth when re-introduced into glioma cell lines. Overexpression of RGC-32 in HeLa cells also delayed progress through G2/M [30]. Subsequent microarray analyses have identified RGC-32 as a gene expressed at low level in multiple myeloma plasma cells and drug resistant glioblastomas and RGC-32 promoter methylation has been shown to correlate with RGC-32 downregulation in non small cell lung cancers [35]–[37]. It is therefore possible that the biological effects of RGC-32 may differ between cell and tumour types and as a result RGC-32 may play duel roles in oncogenesis and tumour suppression. Interestingly, two regulators of RGC-32, TGF-β and RUNX1 also appear to promote or repress tumourigenesis depending on the cell context, developmental stage or tumour stage (for reviews see [58] and [59]) and it is possible that some of the downstream effects of these factors may be mediated through regulation of RGC-32 expression. Our results also highlight the fact that although a number of microarray analyses have implicated RGC-32 downregulation in tumour development, gene expression analyses of this type should be treated with caution until it is formally proven that these RGC-32 mRNA expression changes result in a change in protein expression.

The RUNX family of transcription factors (RUNX1, 2 and 3) play key roles in many developmental processes including hematopoiesis, osteogenesis and neurogenesis [60]. Rat RGC-32 was identified as a RUNX1 target and endogenous mouse RGC-32 has been shown to be upregulated when either RUNX1, 2 or 3 are overexpressed in NIH 3T3 cells [42]–[44]. We now demonstrate that RUNX1 activates RGC-32 transcription in human B-cell-lines. RUNX1 and RUNX3 expression is inversely related in human B-cell lines due to repression of RUNX1 transcription by RUNX3 through direct interaction with sites in the RUNX1 promoter [41]. EBV infected latency III cells that express the EBV transcriptional regulator EBNA 2 display high levels of RUNX3, due to activation of RUNX3 transcription by EBNA2, and corresponding low levels of RUNX1 [40]. By contrast, EBV infected latency I cell-lines that do not express EBNA 2 have high RUNX1 and low RUNX3 expression. Our results provide further support for the differential roles of RUNX1 and RUNX3 in human B-cell lines since RGC-32 mRNA levels mirror those of RUNX1, but not RUNX3. Our results are in contrast to those in NIH 3T3 cells where all three RUNX family members upregulated mouse RGC-32 mRNA expression. It is clear that the differential roles of RUNX1 and RUNX3 are cell-type and context dependent since overexpression of RUNX1 or RUNX3 in NIH3T3 cells provided a survival advantage under stress [44], but RUNX 1 and not RUNX3 expression in EBV infected LCLs blocks cell growth [61]. Since RUNX1c overexpression in transient reporter assays resulted in only approximately 2-fold increases in RGC-32 promoter activity, it is possible that other, as yet unidentified transcription factors, may contribute to RGC-32 mRNA upregulation in latency I cells or that additional regulatory elements not included in the reporter construct play an additional role in transcriptional activation. Nonetheless, endogenous overexpression of RUNX1c resulted in a 1.75-fold increase in RGC-32 mRNA expression, supporting the role for RUNX1c in regulating RGC-32 transcription.

We have identified a novel control point for regulating RGC-32 expression involving translational mechanisms. RGC-32 mRNA is associated with polysomes even when not translated in latency I cells, implicating a post-initiation mechanism for the control of RGC-32 translation. Since miRNAs are known to regulate translational elongation these results raise the possibility that miRNAs may play a role in the regulation of RGC-32 expression. Interestingly a number of cellular miRNAs have been previously shown to be downregulated in EBV positive latency III cells compared to EBV negative and latency I cells, a pattern expected for a miRNA that blocks RGC-32 protein expression in latency I but not latency III cells [62]. It will be interesting to test whether the cellular miRNAs downregulated in latency III cells can repress RGC-32 expression.

Additional clues to mechanisms that could contribute to the inhibition of RGC-32 translation in latency I cell-lines come from studies on the potential functional homologue of RGC-32, the atypical CDK activator, RINGO. RINGO is expressed at the mRNA level in G2 arrested *Xenopus* oocytes but RINGO protein is undetectable due to translational repression by the RNA-binding protein Pumilio-2 (PUM2) Pumilio-2 binds human PUM2 binding element 1 (hPBE1, UNUUANNUGUA) or the human PUM2 binding element 2 (hPBE2, UAUANNUAGU) [63]. We have identified a consensus hPBE2 element in the RGC-32 3′UTR implicating PUM2 as a potential regulator of RGC-32 translation. Previous studies using RNA immunoprecipitation (RIP) techniques to identify PUM2 or PUM2/DAZL co-associated mRNAs have not detected PUM2 association with RGC-32 transcripts in HeLa S3, HEK293 or testis mRNA samples [63]-[65]. Since our studies point to B-cell specific mechanisms for the control of RGC-32 translation, it will be interesting to determine whether PUM2 is able to bind RGC-32 mRNA differentially in EBV-negative and EBV-positive latency I and latency III B-cell-lines.

In summary, we have identified RGC-32 as a key cellular gene that may play a role in promoting the survival and proliferation of EBV-infected cells through deregulation of the G2/M cell-cycle checkpoint. To further investigate the role of RGC-32 in EBV-mediated tumourigenesis, it will be interesting to determine whether RGC-32 expression levels are elevated in EBV-associated post-transplant lymphomas that display the latency III pattern of gene expression and whether RGC-32 protein expression in required for the proliferation of EBV-infected latency III cells. Our studies also reveal that novel post-initiation mechanisms control RGC-32 protein expression in EBV infected B-cells. Since these mechanisms may also control RGC-32 protein expression in other cell-types and tumour tissues, our work highlights the need to perform RGC-32 expression analyses at the protein as well as RNA level.

## Materials and Methods

### Plasmid construction

To create pFLAG RGC-32, RGC-32 was amplified from BJAB E3C-4 [66] cDNA, using primers designed to introduce 5′ XbaI and 3′ BamHI sites (supplementary Table S1), and cloned into pFLAG-CMV-2 (Sigma). pFRT RGC-32 was created by excising the FLAG-RGC-32 sequence from pFLAG RGC-32 as a Sac1/Sma1 fragment, removing the Sac1 overhang using mung bean nuclease and ligating into EcoRV-digested pcDNA5/FRT (Invitrogen). pRGC-32pluc was generated by amplifying a 1.25 kb fragment of the RGC-32 promoter (–1177 to +79 relative to the predicted transcription start site) from genomic DNA, using primers designed to introduce 5′ KpnI and 3′ HindIII sites (supplementary Table S1), and cloning into pGL2-Basic (Promega). pET-RGC-32 was generated by excising the RGC-32 sequence from pFLAG-RGC-32 as a *Sal*I/*Bam*HI fragment and ligating into pET16b (Novagen) digested with XhoI/BamHI.

### Cell lines and culture

The DG75 or BJAB FRT and FRT-RGC-32 cell lines were generated using the Flp-In system (Invitrogen). EBV-negative BJAB B-cell lymphoma cells [67] or DG75 Burkitt's lymphoma cells [68] were transfected with 10 µg linearised pFRT/lacZeo (Invitrogen) via electroporation as described previously [69] to create a stable Flp-In host cell line (BJAB/DG75 FRT). After 48 h Zeocin was added to a final concentration of 400 µg/ml and cells were diluted and aliquoted into 96-well plates to select single cell clones. Genomic DNA was isolated from Zeocin-resistant clones and Southern blot analysis (using a fragment of the lacZ gene as a probe) was performed to determine the number of integrated FRT sites. Cell lines containing single integrants were then screened for beta-galactosidase activity and the line with the highest expression level was stably transfected with 1.8 µg pOG44 (Invitrogen) and 0.2 µg pFRT RGC-32 plasmid using Amaxa nucleofection (kit T, programme T-016). Cells were diluted in media containing hygromycin B (200 µg/ml) 48 h after transfection and cultured to obtain the isogenic hygromycin-resistant cell-lines BJAB FRT-RGC-32 and DG75 FRT RGC-32. The BJAB and DG75 FRT and FRT RGC-32 cell lines were routinely cultured in the presence of 100 µg/ml Zeocin or 200 µg/ml hygromycin B respectively.

The EBV-positive latency I and III Burkitt's lymphoma cell lines Mutu I (cl 179) and Mutu III (cl 48) [39], the IB4 LCL [70] and most EBV negative and positive cell-lines not previously described [69] were provided by Prof. Martin Rowe. The EBV-negative Burkitt's lymphoma cell lines BL2 and BL31 and their EBV BAC infected derivatives were provided by Prof. Martin Allday [15]. LCL#3 was provided by Dr Alison Sinclair [71]. All cell-lines were passaged twice-weekly and cultured using previously described conditions.

### Centrifugal elutriation and flow cytometry

Centrifugal elutriation (Beckman J6-MC centrifuge) was used to separate the different cell-cycle phases as described previously [72] . Mutu III cells were injected in a JE-5.0 rotor with a large separation chamber at 1500 rpm and a flow rate of 30 ml per minute controlled with a Cole-Palmer Masterflex pump. The rotor speed was kept constant and fractions were collected at increasing flow rates (35 ml per minute to 100 ml per minute). The DNA content of the 40–80 ml fractions was determined by propidium iodide staining and flow cytometry (Becton Dickinson) using standard procedures.

### Nuclear/cytoplasmic fractionation

3×10^7^ cells were gently resuspended in 1 ml of lysis buffer B [73] (10 mM Tris pH 8.4, 140 mM NaCl, 1.5 mM MgCl_2_, 0.5% NP40, 1 mM DTT and 200U/ml RNasin (Promega)) and the cytoplasmic supernatant obtained by centrifugation at 2500 rpm for 3 minutes at 4°C using a Sorvall Legend RT centrifuge. The nuclear pellet was resuspended in 1 ml lysis buffer B containing 1/10 volume (100 µl) of detergent (3.3% [wt/vol] sodium deoxycholate and 6.6% [vol/vol] Tween 40) under slow vortexing and the sample incubated on ice for 5 minutes. The nuclear material was re-pelleted and then rinsed with 1 ml of lysis buffer B followed by final pelleting of the nuclear fraction. The supernatant was discarded. 1 ml of TriReagent (Sigma) was then added to both nuclear and cytoplasmic fractions and RNA isolated. For SDS-PAGE, 1 ml of 1× GSB [74] was added to the nuclear fraction and the sample sonicated and 20 µl of the cytoplasmic fraction was mixed with 5 µl 5× GSB.

### RNA half-life determination

Cells were diluted to 4×10^5^/ ml 24 h prior to treatment with a 2 µM sub-toxic dose of actinomycin D (Sigma). Cells were harvested after 0.5, 1, 2 and 4 h.

### Proteasome inhibition

Cells were diluted to 3×10^5^/ ml 24 hrs before the experiment. The proteasome inhibitor MG132 was added to a final concentration of 50 or 100 µM and cells were harvested after 2 or 6 hours for SDS-PAGE analysis.

### Transfection

For RUNX1 overexpression, 5×10^6^ IB4 cells in exponential growth were transfected with 3 µg pCEP4 or pCEP4-RUNX1c [61] (provided by Prof. Paul Farrell) using Amaxa kit T, programme A-023 according to the manufacturer's instructions. 24 hrs post-transfection, cells were selected in 200 µg/ml Hygromycin B and harvested 6 days post-transfection.

For RGC-32 promoter reporter assays, the EBV-negative Burkitt's lymphoma cell-line DG75 was electroporated with plasmid DNA at 230 V and 950 µF (BioRad Gene Pulser II) and luciferase assays carried out as described previously [69]. Cells were transfected with the RGC-32pLuc reporter and pRL-CMV (Promega) as a transfection control, in the absence or presence of pBK CMV RUNX1c (provided by Prof. Paul Farrell).

### DNA damage and flow cytometry

Cells were diluted 24 hours prior to DNA damage and then cultured in the presence of etoposide (Sigma) for 24 or 48 h. Cells were fixed in 100% ethanol and 1×10^6^ cells were resuspended in 500 µl propidium iodide solution (0.1 mg / ml propidium iodide (Sigma) in PBS containing 0.1% Triton X-100) followed by the addition of 12 µl of 2 mg/ml RNase A (Invitrogen). Cells were stained for 30 mins and cell cycle distribution was then analysed using a FACsCaliber Flow Cytometer (BD Biosciences).

### RGC-32 protein preparation

2 litres of *E.coli* BL21 *plysS* cells transformed with pET-RGC32 were induced to express His-RGC-32 by treatment with 1 mM IPTG for 4 h at 37°C. Cell pellets were resuspended in 80 ml of cold buffer A (40 mM PO_4_ buffer pH 7.5, 300 mM NaCl, 2 mM benzamidine, 20 mM Imidazole, 3.5 mM β-mercaptoethanol, protease inhibitor cocktail tablet (Roche)) and freeze-thawed 3 times. 10 µg/ml of DNase I was added and the lysate incubated with rotation for 15 min at room temperature. Lysates were then sonicated for 6×10 s and the insoluble material pelleted by centrifugation at 9.8 K rpm for 20 min in a Sorval SS-34 rotor at 4°C and then washed by resuspension in 20 ml buffer X (50 mM HEPES (KOH) pH 7.5, 10 % glycerol, 2 mM benzamidine, 1 M GuHCl). Brief sonication, centrifugation and washing steps were repeated prior to denaturation of the protein pellet using 20 ml buffer Y (50 mM HEPES (KOH) pH 7.5, 10 % glycerol, 2 mM benzamidine, 6 M GuHCL). Samples were then re-sonicated briefly and the remaining insoluble debris removed by centrifugation. The supernatant was then added to 0.5 ml of NTi agarose resin (Sigma) and mixed by rotation for 90 min at 4°C. Proteins bound to the beads were then gradually refolded by washing twice in 25 ml buffer A, twice in buffer B (40 mM PO_4_ buffer pH 7.5, 300 mM NaCl, 2 mM benzamidine, 20 mM Imidazole, 3.5 mM β-mercaptoethanol, protease inhibitor cocktail tablet, 1% NP40), twice in buffer C (40 mM PO_4_ buffer pH 7.5, 1 M NaCl, 2 mM benzamidine, 20 mM Imidazole, 3.5 mM β-mercaptoethanol, protease inhibitor cocktail tablet, 1% NP40) and twice in buffer A. Protein was eluted three times using 1 ml elution buffer (40 mM PO_4_ buffer pH 7.5, 300 mM NaCl, 100 mM EDTA) and dialysed to remove EDTA.

### Immunoblotting

SDS-PAGE and immunoblotting were carried out as described previously [69], [75]. The following antibodies were used for immunoblotting: anti-actin 1/5000 (A-2066, Sigma), anti-CDK1 1/5000 (Cdc2 p34 sc-54), anti-cyclin B1 1/5000 (sc-245, Santa Cruz Biotechnology), anti-RUNX1 1/40 (Ab-2, Calbiochem), anti-Spt16 1/500, (sc-28734, Santa Cruz Biotechnology) and anti-p53 1/1000 (DO.1, Santa Cruz Biotechnology). RGC-32 was detected using polyclonal rabbit sera raised by Eurogentec against recombinant His-RGC-32.

### cDNA preparation

For analysis of cell-line panels, cells were diluted to 2×10^5^/ ml, harvested after 3 days and total RNA extracted using TriReagent (Sigma). RNA samples were purified using the RNeasy kit (Qiagen) and cDNA synthesised using the ImProm II reverse transcription system and random oligonucleotides (Promega). For RNA half-life experiments, cDNA was prepared from 1×10^5^cells using Power SYBR Green Cells-to-CT Kit (Applied Biosystems).

### PCR

Quantitative PCR (Q-PCR) was performed in duplicate generally using the standard curve absolute quantification method on an Applied Biosystems 7500 real-time PCR machine as described previously [75], and primers for RGC-32, RUNX1c, GAPDH or actin (supplementary Table S1). For PCR across exons 2-4 of RGC-32, cDNA was amplified using Taq polymerase (New England Biolabs) according to the manufacturer's recommended conditions and 35 cycles of 94°C for 30 s, 55°C for 30 s and 72°C for 1 min. For RNA half-life experiments, Q-PCR was carried out using Power SYBR Green Cells-to-CT Kit (Applied Biosystems) and RGC-32 or GAPDH specific primers followed by analysis using the Relative Quantification (ddCt) method.

### Kinase assays

Assays were carried out using the cdc2 kinase assay kit (Upstate) and samples were analysed as described previously [75]. Assays contained up to 5 µM His-RGC-32 protein and 2 units of recombinant CDK1 (cdc2)/Cyclin B1 (NEB).

### Northern blotting

Northern blotting was carried out essentially as described previously [76] using 16 µg total cellular RNA isolated using TriReagent (Sigma). RGC-32 transcripts were detected by hybridization to a 360 bp XbaI/BamHI fragment from pFLAG-RGC-32 containing the complete RGC-32 cDNA, labelled with [α^32^P-dCTP] using the ready to go DNA labelling bead kit (Amersham). Blots were washed 3×10 mins at room temperature in 3× SSC, 0.1% SDS, 1×10 mins in 0.5xSSC, 0.1% SDS and 2×10 mins in 3× SSC, 0.1% SDS at 65°C. Blots were stripped by boiling in water and re-hybridized to a GAPDH probe generated from a 1.3 kb EcoRI fragment from pBSK+GAPDH.

### Polysome analysis

Sucrose gradient density centrifugation analysis was carried out essentially as described previously but in the absence of heparin [77]. Briefly, 3×10^7^ cells in exponential growth were resuspended in fresh growth media at a concentration of 5×10^5^ cells/ml and cultured for 1.5 hrs prior to analysis. Cycloheximide was then added to a final concentration of 0.1 mg/ml for 5 min at 37°C and the cells rapidly cooled in an ice bath. Following two washes in PBS containing cycloheximide, cells were lysed in 500 µl polysome extraction buffer and debris and nuclei removed prior to loading on a 10–60% sucrose gradient as described [77]. Gradients were sedimented at 38,000 rpm for 2 hrs in a SW40 Ti rotor at 4°C. Gradient samples were collected as 0.5 ml or 1 ml fractions by pumping 70% sucrose into the bottom of the gradient and collecting from the top with continuous monitoring at 254 nm. RNA was extracted from fractions by either the addition of 10 µl 10% SDS, 25 µl 0.5 M EDTA and 4 µl 20 mg/ml Proteinase K for 1 hr at 37°C, followed by purification using the RNeasy kit protocol (Qiagen) from step 4, or collecting fractions directly into 3 mls 8 M guanidine hydrochloride and processing as previously described [77]. cDNA was then prepared and transcript levels determined using Q-PCR analysis.

## Supporting Information

Figure S1 **Cell-cycle profiles of elutriated cell fractions.** Mutu III cells were separated into cell-cycle fractions by centrifugal elutriation and a sample of each fraction analysed to determine the cell-cycle phase using propidium staining of DNA followed by flow cytometry. The cell-cycle phases attributed to the majority of cells in each fraction based on DNA content are indicated.
(PDF)

Table S1 **Sequences of primers used for conventional and Q-PCR.**
(PDF)
