# Supplementary figures and images for: Upregulation of the Cell-Cycle Regulator RGC-32 in Epstein-Barr Virus-Immortalized Cells
Source: PLoS One. 2011 Dec 6;6(12):e28638. doi: 10.1371/journal.pone.0028638 (PMC3232240; doi:10.1371/journal.pone.0028638)

Mutu III cells

A

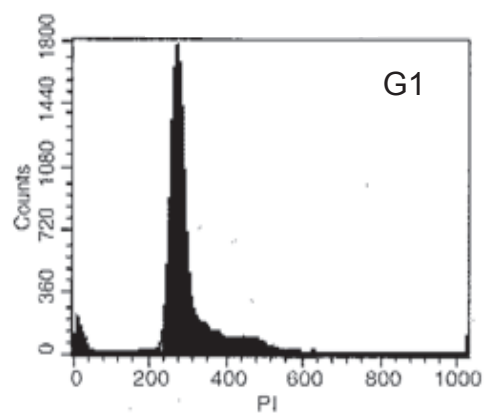

B

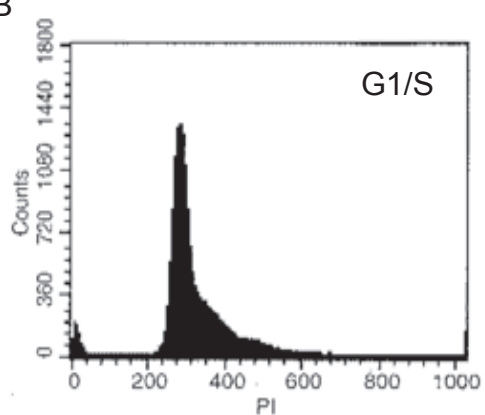

C

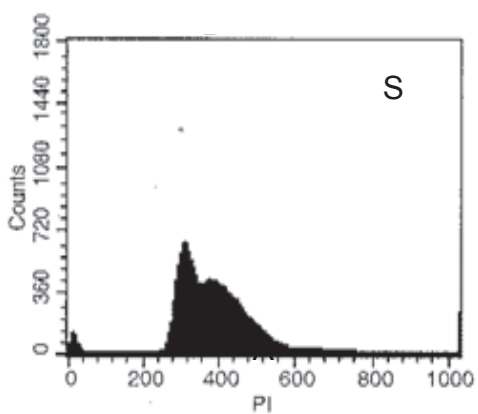

D

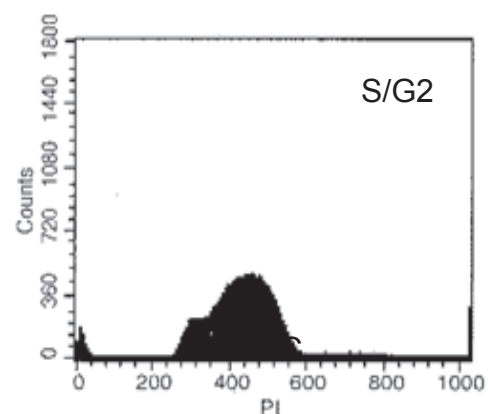

E

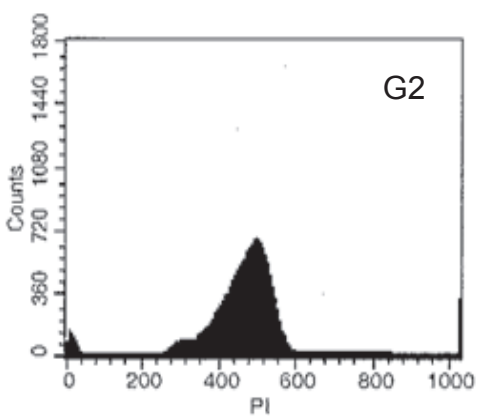

F

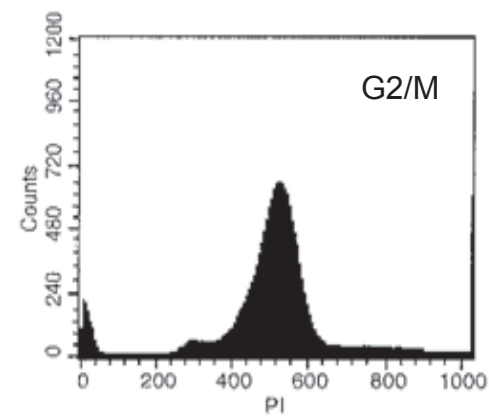

Supplement: Figure S1 — Cell-cycle profiles of elutriated cell fractions. Mutu III cells were separated into cell-cycle fractions by centrifugal elutriation and a sample of each fraction analysed to determine the cell-cycle phase using propidium staining of DNA followed by flow cytometry. The cell-cycle phases attributed to the majority of cells in each fraction based on DNA content are indicated. (PDF) [file pone.0028638.s001.pdf]
