# Supplementary material for: Upregulation of the Cell-Cycle Regulator RGC-32 in Epstein-Barr Virus-Immortalized Cells
Source: PLoS One. 2011 Dec 6;6(12):e28638. doi: 10.1371/journal.pone.0028638 (PMC3232240; doi:10.1371/journal.pone.0028638)
Supplement: Table S1 — Sequences of primers used for conventional and Q-PCR. (PDF) [file pone.0028638.s002.pdf]

Name	Sequence
pFLAG RGC-32	
Forward MW111	GCTCTAGAATGAAGCCGCCCGCGGAG
Reverse MW112	CGCGGATCCCTATCACATACTTGCTAAAGTTTTGTCAAG
pRGC-32pluc	
Forward MW106	CGGGGTACCGACAAGCATATCCCTAGG
Reverse MW107	CCCAAGCTTGCTTGCTGTCCCGCACAC
RGC-32 exon 2-4	
Forward MW391	CCACTTCCACTACGAGGAGCA
Reverse MW392	TGTCAAGATCAGCAATGAAGGCT
RGC-32 exon 3 (Q-PCR)	
Forward MW86	TTATAGGAACAGCCTCAGCTTC
Reverse MW87	CTGAGGAGTGACAGTGGCAG
RGC-32 exon 2-3 (Q-PCR)	
Forward MW385	CCAGTGTCAGCGACAGCA
Reverse MW386	GGTAGAGTCTGTTGGAGAATTCAGT
RGC-32 exon 4-5 (Q-PCR)	
Forward MW387	AGCCTTCATTGCTGATCTTGA
Reverse MW388	GCAGGTCCTCGGAACTTTCT
GAPDH (Q-PCR)	
Forward MW84	TCAAGATCATCAGCAATGCC
Reverse MW85	CATGAGTCCTTCCACGATACC
Actin (Q-PCR)	
Forward MW417	CTGGCACCACACCTTCTACA
Reverse MW418	TAGCACAGCCTGGATAGCAA
RUNX 1c (Q-PCR)	
Forward MW349	AACCACAGAACCACAAGTTGG
Reverse MW350	TTGCATTCAGTGTGATTCGTC

Table S1. Sequences of primers used for conventional and Q-PCR. All sequences are shown 5’ to 3’.
